# Enzymatic Modification of Potato Starch: Starch-Acting Enzyme Resources, Structure–Function Relationships and Value-Added Applications

**DOI:** 10.3390/foods15193497

**Published:** 2026-09-29

**Authors:** Shigui Li, Chenxi Dong, Jiangwei Yang, Xiao Wang, Jinchao Yue, Zixuan Wang, Zhen Yang, Shiyu Wei

**Affiliations:** 1 College of Life Science and Technology, Gansu Agricultural University, Lanzhou 730070, China; yjw@gsau.edu.cn; 2 Gansu Provincial Key Laboratory of Microbial Resources Utilization, School of Biological and Pharmaceutical Engineering, Lanzhou Jiaotong University, Lanzhou 730070, China; dongchenxiii@outlook.com (C.D.); yjcstudy2026@outlook.com (J.Y.); 20243701118@stu.lzjtu.edu.cn (Z.W.); 20243701120@stu.lzjtu.edu.cn (Z.Y.); 3 College of Agronomy, Gansu Agricultural University, Lanzhou 730070, China; 1073323010101@st.gsau.edu.cn

**Keywords:** potato starch, enzymatic modification, 4-α-glucanotransferase, branching enzyme, resistant starch, structure–function relationship

## Abstract

Potato (*Solanum tuberosum* L.) starch is valued for its large granules, high paste viscosity, and neutral taste, yet its gelatinized form is rapidly digested and its native granules resist enzymatic attack. This review synthesizes peer-reviewed studies indexed in Scopus, Web of Science, PubMed, Crossref, and Google Scholar from January 2000 to September 2026, with emphasis on 2018 onward, classifying evidence as direct (potato starch as substrate) or extrapolated (other starch systems). Hydrolases depolymerize potato starch, generate porous granules, or supply linear chains for type 3 resistant starch, whereas 4-α-glucanotransferases, branching enzymes, amylosucrase, and cyclodextrin glucanotransferases deplete amylose, elongate exterior chains, introduce α-1,6 linkages, or produce cyclic glucans. The strongest direct evidence supports thermoreversible gels, pullulanase-assisted resistant starch, slowly digestible starch, low-digestibility starch, cyclodextrin production, and acid-assisted porous starch. Ultrasound, high hydrostatic pressure, irradiation, and hydrothermal treatments modulate enzyme accessibility but remain strongly condition-dependent. Translation is constrained by gelatinization and drying energy, reaction viscosity, enzyme cost and recovery, and heterogeneous digestibility assays. Priority actions include raw-granule-active enzymes, standardized potato-starch assays, rational enzyme cascades, structure-guided enzyme engineering, and product-specific human and life-cycle assessments.

## 1. Introduction

Potato ranks as the world’s fourth most important food crop after maize, wheat, and rice [1], with starch serving as the principal storage carbohydrate in its tubers. Potato starch is produced industrially on a large scale, particularly in Europe and China, and is widely used in food, paper, textile, adhesive, and pharmaceutical applications due to its large granule size, high paste viscosity, low protein and lipid content, and neutral taste [2,3]. Despite these advantages, native potato starch has intrinsic drawbacks: its pastes lose viscosity under shear and acidic conditions; its gels undergo retrogradation and syneresis during storage; and its gelatinized form is rapidly and almost completely digested, resulting in a high glycemic response [4,5]. Therefore, modification of the starch molecule is necessary to tailor its functionality for specific applications.

For decades, the industry has relied on chemical derivatization—such as cross-linking, oxidation, acetylation, and hydroxypropylation—to achieve these goals. However, these reagents can leave residues, generate by-product streams, and, for food use, require additive declaration in many jurisdictions [5,6]. Enzymatic modification offers an attractive alternative: starch-active enzymes operate under comparatively mild aqueous conditions, exhibit high regio- and stereospecificity, and introduce no deliberately added chemical substituents. Nevertheless, “enzyme-treated” is not automatically equivalent to “additive-free” or “clean-label” in every jurisdiction; the legal status depends on the enzyme, the treatment, the final function, and local additive and processing-aid regulations [7,8,9,10]. The commercial use of amylomaltase-treated potato starch as a gelling, thickening, and fat-replacing ingredient demonstrates that enzyme-modified potato starch is an industrial reality rather than merely a laboratory concept [11,12].

Potato starch is a particularly instructive substrate for enzymatic modification. Its B-type crystalline packing, long amylopectin branch chains, and high phosphate monoester content render its native granules markedly more resistant to amylolytic attack than cereal A-type starches, while these same features make its gelatinized form highly responsive to chain-length and linkage engineering [3,13]. Consequently, the outcome of enzymatic treatment of potato starch depends on the enzyme chosen, the physical state of the substrate, enzyme dosage, reaction time, and treatment sequence. Potato starch is therefore an effective model for examining the structure–function relationships of starch-active enzymes [14,15].

The nutritional terms used throughout this review follow the Englyst classification: rapidly digestible starch (RDS) is hydrolyzed within 20 min; slowly digestible starch (SDS) is hydrolyzed between 20 and 120 min; and resistant starch (RS) is not hydrolyzed within 120 min under the specified conditions. RS is further divided into RS1 (physically inaccessible starch), RS2 (native resistant granules such as raw high-amylose or raw potato starch), RS3 (retrograded amylose or amylopectin), RS4 (chemically modified starch), and RS5 (amylose–lipid complexes) [16,17]. “Retrogradation” denotes the reassociation of gelatinized starch chains, whereas “gel” refers to the macroscopic network formed by a sufficiently concentrated starch system. “Gelatinization” denotes the loss of granule crystalline order during heating in water, and “starch paste” refers to the hydrated dispersion obtained after gelatinization.

Several reviews have covered starch-converting enzymes in general [18], enzymatic approaches for structuring starch [5], and individual enzyme families such as the 4-α-glucanotransferases [19,20] and the 4,6-α-glucanotransferases [21]. These valuable reviews are generally organized around an enzyme family, a reaction type, or starch modification as a whole. The added value of the present review lies in its substrate-centered organization around potato starch, its explicit separation of direct potato-starch evidence from mechanistic evidence obtained with other starches, and its decision-oriented treatment of substrate physical state, enzyme class, fine-structure changes, functionality, and process constraints. The novelty claim is therefore not that potato starch has never appeared in a review, but that potato starch is used here as the primary organizing variable for enzyme selection and product design.

### Review Methodology and Evidence Classification

Database searches were performed on 10 September 2026 using Scopus, Web of Science Core Collection, PubMed, Crossref, and Google Scholar. The core search string combined “potato” or “*Solanum tuberosum*” with “starch” and terms related to enzymatic modification, including “enzyme,” “amylase,” “glucoamylase,” “pullulanase,” “isoamylase,” “glucanotransferase,” “branching enzyme,” “amylosucrase,” “cyclodextrin glucanotransferase,” “phosphorylase,” “hydrolysis,” “gel,” “resistant starch,” “slowly digestible starch,” “digestibility,” and “porous starch.” The main search period covered January 2000 to September 2026, with emphasis on 2018–2026. Earlier publications were included when identified as foundational works or through backward citation chasing. English- and Chinese-language peer-reviewed articles and authoritative regulatory or statistical sources were eligible. Patents, conference abstracts, studies without accessible full texts, and studies that did not identify the starch source were excluded.

Because the outcomes of interest span molecular structures, rheology, granular morphology, in vitro digestion, and industrial products, this article is a structured narrative review rather than a PRISMA systematic review. Therefore, we do not claim an exhaustive effect-size synthesis or a single pooled performance ranking. Every key result is labeled as “direct” when potato starch was the experimental substrate and “extrapolated” when another starch was used but the mechanism is relevant to potato starch. This distinction is particularly important for porous starch morphology, enzyme subsite engineering, hydrothermal treatments, nanoparticles, and some regulatory interpretations.

## 2. Structural Determinants of Potato Starch That Shape Enzymatic Modification

### 2.1. Granule Architecture and B-Type Crystallinity

**Figure 1:**
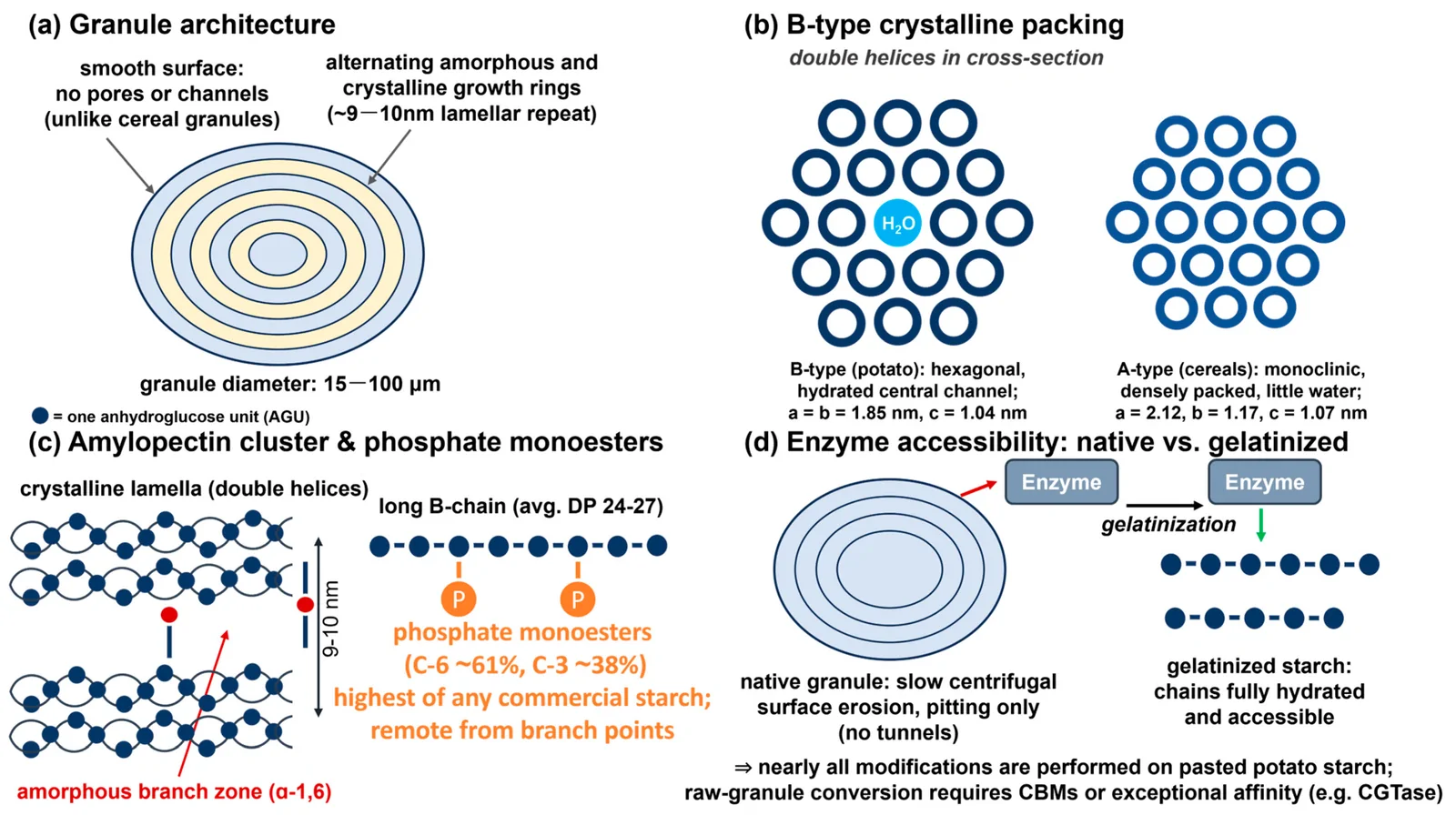
Multiscale structural determinants of potato starch and their consequences for enzymatic attack. Throughout the figure, each navy sphere denotes one anhydroglucose unit (AGU). (**a**) Granule architecture: large, smooth-surfaced granules (15–100 μm) with alternating amorphous and crystalline growth rings [27]. (**b**) Hexagonal, hydrated B-type packing of amylopectin double helices (open cylinders in cross-section) around a central water channel (unit cell a = b = 1.85 nm, c = 1.04 nm, 36 H_2_O per unit cell), contrasted with the denser monoclinic A-type polymorph of cereal starches (a = 2.12 nm, b = 1.17 nm, c = 1.07 nm, γ = 123.5°), as established in classical crystallographic studies [28,29]. (**c**) Amylopectin cluster organization: crystalline lamellae of double helices (~9–10 nm repeat), amorphous α-1,6 branch zones, and long B-chains (average DP 24–27) carrying phosphate monoesters on C-6 and C-3 hydroxyls remote from branch points [27,30]. (**d**) Enzyme accessibility: native granules undergo only slow centrifugal surface erosion, whereas gelatinization renders the chains fully hydrated and accessible to enzymes.

Starch accumulates within the amyloplasts of potato tubers as water-insoluble, semi-crystalline granules characterized by alternating amorphous and crystalline growth rings (Figure 1a), which are spaced approximately 9–10 nm apart [22,23]. Potato starch granules are among the largest of all commercially utilized starches, with diameters ranging from approximately 15 to 100 μm. These granules exhibit an oval to spherical morphology and possess a smooth surface that is largely devoid of the channels and surface pores commonly observed in maize and sorghum granules [22,24]. At the molecular level, the granule comprises amylose, a sparsely branched α-1,4-glucan of intermediate molecular weight, and amylopectin, which constitutes the major fraction (typically 69–80% in normal potato starch). The unit chains of amylopectin are organized into clusters of double helices (Figure 1c) [3,23]. Notably, potato amylopectin is characterized by an unusually high proportion of long B-chains, with an average degree of polymerization (DP) of approximately 24–27, compared to DP 18–23 in cereal amylopectins. These extended chains form double helices that pack into the hexagonal, highly hydrated B-type allomorph (Figure 1b), a structural feature typical of tuber starches [23,25]. The B-type lattice contains a central water-filled channel per 36 glucosyl residues and exhibits greater resistance to hydration-induced swelling at moderate temperatures and to enzymatic penetration than the densely packed A-type polymorph found in cereal starches [25,26]. These structural characteristics underpin a fundamental dichotomy in starch processing: native potato starch granules are resistant to modification, yet they become highly responsive following gelatinization. Consequently, the selection of enzymes and physical treatments must consider the physical state of the substrate as a primary factor, rather than focusing solely on the desired final functionality.

### 2.2. Phosphate Monoesters and Minor Granule Components

A distinguishing characteristic of potato starch is its high content of covalently bound phosphate monoesters, which is the highest among commercially available starches. On average, potato amylopectin contains one phosphate group per 200–400 glucosyl units, predominantly esterified at the C-6 (approximately 61%) and C-3 (approximately 38%) hydroxyl groups of long B-chains, located distal to branch points (Figure 1c) [22]. These negatively charged phosphate groups generate electrostatic repulsion within the crystalline lamellae, induce local packing defects, and significantly enhance granule hydration, swelling power, paste viscosity, and paste clarity [3].

Comparative analyses of potato starches with low and high phosphate content have demonstrated that increased phosphate levels disrupt the granular matrix, reduce the relative abundance of double helices and short-range order at the granule surface, and decrease gelatinization enthalpy [3]. The role of phosphate monoesters in directly enhancing enzymatic accessibility remains unconfirmed as an independent causal factor in potato starch. This interpretation is primarily inferential: phosphate groups are concentrated in long-chain, amorphous regions; granules with high phosphate content exhibit greater surface disorder and diminished short-range order; and certain enzymes, notably cyclodextrin glycosyltransferase (CGTase), can degrade raw potato granules. Consequently, it is more precise to assert that phosphate content correlates with hydrated and partially disordered regions that may facilitate enzyme access, rather than to claim that phosphate has been definitively shown to accelerate the activity of all enzyme classes [3,31].

Potato starch contains substantially less internal lipid than cereal starches, and its native granule surface is characterized by relatively low protein content. This composition reduces amylose–lipid complex formation during mild processing; however, the addition of lipids, emulsifiers, or other processing ingredients can still promote the formation of RS5-type complexes, thereby influencing enzyme accessibility. The literature specific to potato starch is considerably more extensive regarding phosphate-related effects than lipid-mediated effects; thus, conclusions concerning lipids are currently regarded as mechanistic hypotheses rather than established, potato-specific phenomena [25,32].

### 2.3. Enzymatic Susceptibility of Native Potato Starch Granules

The resistance of native potato starch granules to enzymatic hydrolysis is well documented. Comparative degradation studies employing α-amylase from *Bacillus amyloliquefaciens* have revealed distinct erosion patterns among botanical sources: whereas corn, rice, and wheat granules are degraded both centrifugally and centripetally, potato granules predominantly undergo slow centrifugal surface erosion characterized by pitting without the formation of well-developed tunnels (Figure 1d) [13]. The outer layers of large granules exhibit inherently greater resistance than the interior regions; consequently, potato and high-amylose starches are hydrolyzed more slowly than waxy cereal starches under comparable conditions [33,34]. This behavior is attributable to the combination of B-type crystallinity, large granule size, long amylopectin branch chains, and the absence of surface pores [13,25].

Accordingly, most enzymatic modifications of potato starch are conducted on gelatinized starch or starch pastes, wherein granule integrity is disrupted and glucan chains are fully hydrated. Although modification of raw granules is feasible, it generally necessitates enzymes with high intrinsic affinity for granular starch or carbohydrate-binding modules. The clearest reported exception among the enzyme classes discussed herein is CGTase from *Bacillus macerans*, which has been shown to degrade intact potato granules and convert them directly into cyclodextrins; electron microscopy indicated that the inner regions of the granule were more susceptible than the outer shell [31]. Nevertheless, this finding should not be generalized to all CGTases or all raw potato starches. Therefore, elucidating which enzymes act upon specific physical states of potato starch constitutes a fundamental structure–function question in this field and provides the framework for the subsequent enzyme-by-enzyme analysis.

At the solid–liquid interface, productive binding to the insoluble granule and subsequent desorption are mechanistically distinct from bond cleavage occurring in solution. Recent investigations of starch-processing glycoside hydrolases have identified interfacial adsorption, surface topology, substrate swelling, and mass transfer as critical determinants of raw-starch conversion efficiency [35]. This conceptual framework accounts for the observed effects of carbohydrate-binding modules and surface-disrupting pretreatments on potato starch hydrolysis, despite the absence of alterations to catalytic residues.

## 3. Hydrolases: Controlled Depolymerization of Potato Starch

### 3.1. α-Amylases

**Figure 2:**
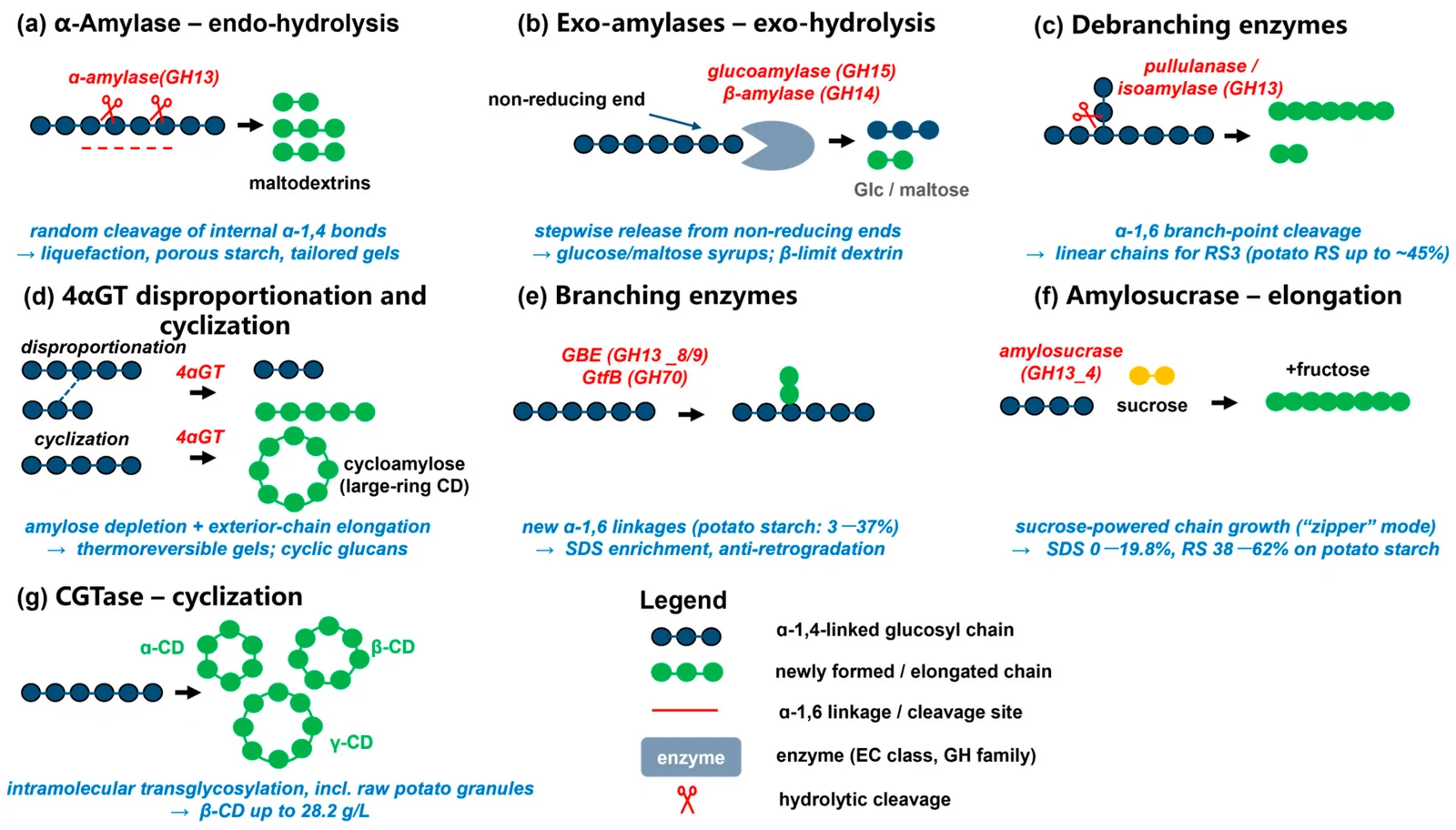
Schematic representation of enzyme actions on potato amylopectin and amylose: (**a**) α-amylase endo-hydrolysis; (**b**) glucoamylase and β-amylase exo-hydrolysis; (**c**) pullulanase/isoamylase debranching; (**d**) 4αGT disproportionation and cyclization; (**e**) GBE/GtfB branching; (**f**) amylosucrase chain elongation; (**g**) CGTase cyclization. The reaction topology and symbol conventions are consistent with established starch enzymology literature [18,20,21,43].

α-Amylases (EC 3.2.1.1) are endo-acting enzymes that catalyze the hydrolysis of internal α-1,4-glucosidic bonds in starch via a retaining double-displacement mechanism (Figure 2a) [36,37]. The majority of α-amylases are classified within the glycoside hydrolase family GH13, with additional members identified in families GH57, GH119, and GH126 [37]. Industrially, thermostable α-amylases derived from *Bacillus licheniformis* and related bacilli serve as principal agents in starch liquefaction, generating maltodextrins that facilitate subsequent saccharification, sweetener production, and fermentation processes [18].

Beyond bulk liquefaction, the specific action pattern of an α-amylase—defined by its relative activity on amylose versus amylopectin and its preference for external versus internal chain cleavage—significantly influences the molecular weight distribution and functional properties of the resulting starch products. This concept is substantiated by a systematic comparison of phylogenetically diverse GH13 α-amylases acting on gelatinized potato starch. Notably, an α-amylase from an uncultured marine bacterium, Um-αAmy, exhibited preferential and controlled degradation of amylose with limited activity on amylopectin; the starch products, enriched in intermediate-length amylose chains, formed strong gels. In contrast, highly efficient thermostable *Bacillus* α-amylases, commonly employed as liquefaction benchmarks, produced starches with poor gelling capacity [15]. The moderate thermal stability of Um-αAmy was advantageous in this experimental context, as it permitted precise termination of the reaction near the desired degree of depolymerization. Multivariate analysis of molecular weight distributions generated by twenty GH13 α-amylases categorized the enzymes into six functional groups, with amylopectin-degrading ability serving as the principal discriminant; structural predictions correlated more open active sites with enhanced amylopectin-degrading activity [15,38]. These findings demonstrate that enzyme identity and active-site accessibility can critically influence product design within the specific potato starch systems examined. However, this should not be construed as a universal assertion that enzyme dosage is inconsequential; rather, dosage and reaction time remain key determinants of the reaction endpoint once an enzyme has been selected. Furthermore, Um-αAmy represents an experimentally characterized candidate enzyme rather than an established commercial liquefaction agent.

When applied to granular potato starch below its gelatinization temperature, α-amylases induce erosion of the granule surface and can enlarge pre-existing micropores. Pore formation in potato starch was enhanced by combining α-amylase or glucoamylase digestion with mild acid hydrolysis, indicating that limited surface weakening can synergize with enzymatic erosion [39]. The generalization that α-amylase produces larger and shallower pores, whereas glucoamylase generates smaller and deeper pores, is primarily derived from studies on non-potato porous starches, particularly corn starch, and is thus considered extrapolated evidence in Table 1. Consequently, enzyme-dependent pore geometry should be experimentally validated for each specific potato starch and processing condition [40].

### 3.2. Glucoamylases

Glucoamylases (EC 3.2.1.3; GH15) are inverting, exo-acting hydrolases that sequentially release β-D-glucose units from the non-reducing ends of α-1,4-linked glucans, exhibiting limited capacity to cleave α-1,6 glycosidic bonds (Figure 2b) [41,42]. The catalytic domain of these enzymes adopts a twisted (α/α)_6_-barrel fold featuring a funnel-shaped active site. Many fungal glucoamylases possess a C-terminal CBM20 starch-binding domain, which is connected via an O-glycosylated linker [41,42]. Glucoamylases derived from *Aspergillus niger*, *A. awamori*, and *Rhizopus oryzae* are widely employed in industrial starch saccharification processes.

The structural significance of the CBM20 domain and its role in raw-starch binding and granular hydrolysis have been primarily elucidated through structural and comparative analyses involving fungal enzymes and cereal or model substrates. Specifically, for potato starch, the most direct evidence of modification involves the combined application of glucoamylase with α-amylase and mild acid hydrolysis to facilitate the formation of porous granules [39]. Additionally, glucoamylase is utilized saccharolytically to remove digestible fractions during resistant starch production and within liquefaction–saccharification sequences.

### 3.3. Debranching Enzymes: Pullulanase and Isoamylase

Pullulanases (EC 3.2.1.41) and isoamylases (EC 3.2.1.68) are glycoside hydrolase family 13 (GH13) enzymes that specifically cleave the α-1,6 glycosidic linkages at the branch points of amylopectin (Figure 2c) [43,47]. Type I pullulanases hydrolyze both pullulan and starch, whereas type II pullulanases additionally cleave α-1,4 bonds. Isoamylases exhibit a preference for the short branch chains of amylopectin over pullulan [43,48]. The debranching of amylopectin results in linear chains capable of reassociating into double helices and subsequently retrograding into enzyme-resistant crystallites, representing a critical step in the production of resistant starch type 3 (RS3) [32].

Three studies—two employing debranching enzymes and one non-debranching comparator—illustrate this process. A cold-active type I pullulanase (PulM), identified from a hot-spring metagenome, exhibited optimal debranching activity at 40 °C and retained over 50% activity at 4 °C. Treatment with PulM increased the apparent amylose content of potato starch by approximately 80%, and retrogradation of the debranched starch yielded about 45% RS3 with enhanced crystallinity and thermal stability [44]. The second study combined α-amylase and pullulanase treatments under Box–Behnken-optimized conditions: α-amylase was applied at pH 6.5 and 70 °C for 15 min at 4 U/g, followed by pullulanase treatment at pH 5.0 and 60 °C for 24 h at 8 U/mL. This protocol produced resistant starch with a yield of 44.48 ± 1.37%, comprising 21.23% rapidly digestible starch (RDS) and 36.32% slowly digestible starch (SDS) as determined by the Englyst method [45]. The third process involved ultrasound-assisted treatment of pre-gelatinized potato starch with a thermostable α-amylase; as this method does not involve debranching, it should not be categorized alongside the pullulanase studies. This approach yielded resistant starch at 14.25% with 19.31% RS content and is more appropriately classified as a physical-field-assisted depolymerization process [49].

Direct enzymatic modification of intact potato starch granules by isoamylase has also been demonstrated. Ulbrich et al. treated commercial potato starch at 40% *w*/*w* concentration and 35 °C for 60 min at pH 4.5, using 100, 250, or 400 μL of a 200 U·mL^−1^ isoamylase preparation per 50 g starch. The granular integrity was largely preserved, and the primary molecular effect was amylopectin debranching. However, subsequent ethanol washing, drying, and grinding also influenced molecular mass, solubility, and gelation behavior. This study demonstrates the feasibility of selective granular debranching but also highlights the necessity of non-thermal enzyme inactivation and appropriate process-blank controls in raw-granule modification protocols [46].

It is important to note that the numerical differences reported across these studies should not be interpreted as a straightforward ranking of enzyme efficacy. Variations in yield, RS content, gelatinization state, retrogradation duration, debranching efficiency, enzyme dosage, and the specific analytical fractions measured represent distinct endpoints. Therefore, standardized reporting of starch concentration, enzyme activity units, reaction stoichiometry, retrogradation conditions, and both yield and RS content is essential for meaningful comparison.

### 3.4. β-Amylase

β-Amylases (EC 3.2.1.2; GH14) are exo-acting enzymes that cleave maltose units from the non-reducing ends of polysaccharide chains (Figure 2b). These enzymes are unable to bypass α-1,6 branch points, resulting in the accumulation of β-limit dextrins. Studies on raw potato granules have demonstrated that β-amylases derived from *Bacillus cereus* and soybean exhibit very slow migration across the granule surface, indicating that B-type granules exhibit greater resistance to exo-acting enzymes compared to endo-acting enzymes [13]. In molecular analyses, controlled β-amylolysis can selectively remove exterior chain stubs up to the first branch point, thereby generating β-limit dextrins that provide valuable information regarding exterior chain length and branch spacing. Consequently, β-amylases are primarily employed as analytical or chain-profiling tools and in maltose-focused processes, rather than as standalone enzymes for modifying potato starch [13,37].

In summary, evidence from hydrolase activity most directly supports mechanisms involving chain scission, debranching, and the formation of RS3 from gelatinized potato starch. However, direct evidence concerning pore geometry in potato starch is limited, and several morphological generalizations remain extrapolated from studies on corn starch systems.

## 4. Glucan Transferases: Reassembling Potato Starch Fine Structure

### 4.1. The Catalytic Landscape of Starch-Active Transferases

Hydrolases function by shortening glucan chains, whereas transferases facilitate their rearrangement. These enzymes operate via retaining double-displacement mechanisms, cleaving α-1,4 glycosidic bonds, stabilizing covalent glycosyl–enzyme intermediates, and subsequently reattaching the glucosyl units to different chains, the same chain, or water. Through these processes, they modulate chain length distribution, generate novel linkage types, or produce cyclic compounds [18,20]. Four principal transferase activities are integral to the modification of potato starch: disproportionation and cyclization catalyzed by 4-α-glucanotransferases (EC 2.4.1.25; GH77, GH13_5, and GH57); branching mediated by glycogen branching enzymes (EC 2.4.1.18; GH13_8/GH13_9) and GH70 4,6-α-glucanotransferases; sucrose-driven chain elongation by amylosucrase (EC 2.4.1.4; GH13_4); and cyclization by cyclodextrin glycosyltransferase (CGTase; EC 2.4.1.19; GH13_2). Additionally, α-glucan phosphorylase is considered separately as a promising emerging enzyme [6,21].

**Figure 3:**
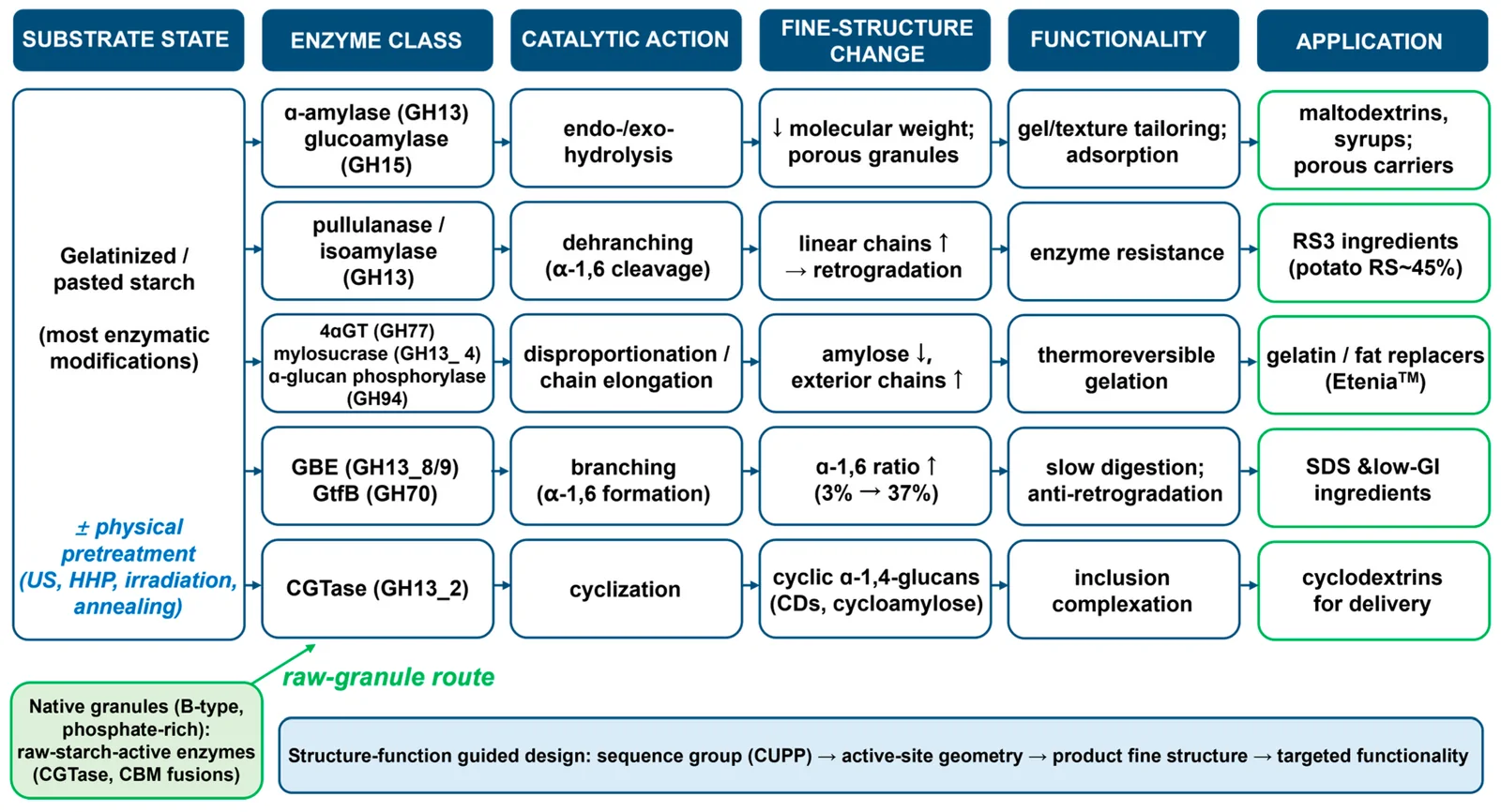
Structure–function roadmap for enzyme selection in the modification of potato starch. The roadmap outlines the progression from substrate physical state to enzyme class, catalytic action, fine-structure alteration, resulting functionality, and eventual application. The third catalytic-action category is “debranching (α-1,6 cleavage),” and the resulting functionality category includes “SDS & low-GI ingredients.” α-Glucan phosphorylase is depicted as an emerging enzymatic opportunity in potato starch modification.

Potato tubers inherently contain a plant 4-α-glucanotransferase, known as the D-enzyme, which has been purified and cloned from potato and is postulated to play a role in starch metabolism [50]. The commercial standard for bacterial transferases is amylomaltase-treated potato starch, marketed as Etenia^TM^ by AVEBE; this product serves as a gelling and texturizing agent produced using *Thermus thermophilus* amylomaltase [11]. The commercial success of this enzyme has stimulated ongoing research aimed at identifying transferases with enhanced thermostability, substrate specificity, and catalytic efficiency [19,20]. The logic that links enzyme choice to product outcome in potato starch modification is summarized as a structure–function roadmap in Figure 3.

### 4.2. 4-α-Glucanotransferases (Amylomaltases, GH77)

4-α-Glucanotransferases catalyze the transfer of α-1,4-glucan segments to the 4-OH group of acceptor glucans or glucose, facilitating reactions such as disproportionation, cyclization, coupling, and hydrolysis (Figure 2d) [20,51]. When applied to gelatinized potato starch, disproportionation predominates: amylose chains serve progressively as donors and are consumed, while amylopectin exterior chains undergo elongation. This process yields an amylose-depleted, long-chain amylopectin that forms a white, opaque gel network exhibiting thermoreversible, gelatin-like properties upon reheating [11,52].

A seminal demonstration of this phenomenon was provided by the hyperthermophilic archaeon *Pyrobaculum aerophilum* IM2. Its amylomaltase (PyAMase; optimal pH 6.7; optimal temperature 95 °C) generated a thermoreversible gel from pasted food-grade potato starch at concentrations of 3% or higher [52]. Approximately half of the amylomaltase-modified potato starch gels melted at 37 °C, with complete melting observed by 60 °C; in contrast to gelatin gels, these gels are susceptible to degradation by salivary amylase [5]. The gel transition temperature exhibited a positive correlation with the presence of long amylopectin chains (degree of polymerization [DP] 60–80) and a negative correlation with short chains (DP 11–21). Nuclear magnetic resonance (NMR) relaxometry revealed that water and chain mobility approached those characteristic of gelatin gels only at elevated polymer concentrations [53,54]. Partial enzymatic conversion resulted in more brittle gels with discontinuous crystalline assemblies, indicating that precise control of conversion is necessary to modulate texture [55].

Recent investigations have advanced the systematic selection of enzymes. Six phylogenetically distinct glycoside hydrolase family 77 (GH77) enzymes, representing four domain architectures, were evaluated on potato starch; four exhibited activity and induced qualitatively similar modifications, although their catalytic rates varied by over two orders of magnitude. Among these, the *Thermus thermophilus* enzyme Tt4αGT demonstrated an optimal temperature of 73 °C and the highest efficacy in modifying potato starch [14]. A comprehensive mining of over 12,000 GH77 sequences using Conserved Unique Peptide Pattern grouping, followed by heterologous expression of 27 representatives, identified *Azospirillum thermophilum* At4αGT and *Thermus* sp. T24αGT as promising candidates, with T24αGT marginally surpassing the Tt4αGT benchmark [56]. Enzymes comprising a single catalytic domain exhibited both high thermostability and substantial starch-modifying capacity within this dataset; however, this observation constitutes a screening criterion derived from a limited sample rather than a definitive structural principle.

The 4αGT from *Thermoproteus uzoniensis* produced a thermoreversible potato starch gel characterized by enhanced rheological properties and improved freeze–thaw stability [57]. Subsequent treatment with amylosucrase further augmented gel strength through re-elongation of exterior chains [58]. By retaining a controlled proportion of amylose rather than complete elimination, recent studies have generated potato starch gels with tunable sol–gel transition temperatures, thereby demonstrating that residual amylose functions as a design parameter rather than merely a substrate to be consumed [59].

### 4.3. Branching Enzymes and 4,6-α-Glucanotransferases

Glycogen branching enzymes (GBEs; EC 2.4.1.18; GH13_8/GH13_9) catalyze the cleavage of an α-1,4 glycosidic bond within a donor chain and subsequently reattach the liberated oligosaccharide to the C-6 hydroxyl group, thereby generating new α-1,6 branch points. The resulting products, characterized by shorter and more densely branched structures, exhibit enhanced resistance to retrogradation and reduced digestibility. GBEs belong to the α-amylase family and possess a (β/α)_8_-barrel fold, frequently incorporating a CBM48 module; the configuration of the extended substrate-binding cleft modulates the length of the transferred donor chain and consequently influences the branching pattern [36,37,60].

The enzymatic outcome is contingent upon both the enzyme variant and the substrate employed. For instance, GBE derived from *Geobacillus thermoglucosidans* increased the proportion of α-1,6 linkages and decreased the in vitro digestibility of granular starches from corn (both normal and waxy varieties), potato, and tapioca to varying degrees, with the most pronounced effects observed in tapioca starch [61]. The amylose content of the substrate significantly affects the branching pattern [60]. Specifically, treatment of potato starch with GBE from *Microvirga* sp. MC18 (McGBE) elevated the α-1,6 linkage content from 3.0% to 6.0%, shifted chain length distribution from degrees of polymerization (DP) greater than 25 toward DP less than 13, inhibited both short- and long-term retrogradation, and increased the slowly digestible starch (SDS) content by 65.8% relative to native potato starch [62].

Sequential enzymatic modification of maize starch, involving initial branching by GBE followed by treatment with 4-α-glucanotransferase (4αGT), yielded densely branched, slowly digestible α-glucans and enhanced curcumin retention in starch–alginate beads. Although this evidence pertains to maize starch rather than potato starch, it exemplifies a cascade strategy—first introducing branches, then redistributing chain lengths—that warrants direct investigation in potato starch due to its inherently long amylopectin chains [63].

In contrast, GH70 family 4,6-α-glucanotransferases (GtfB) facilitate α-1,6 linkage formation via a mechanistically distinct pathway. These enzymes cleave α-1,4 linkages and transfer glucosyl segments to the C-6 position, synthesizing either linear isomalto-/malto-oligosaccharides or short α-1,6 branches depending on the enzyme subtype [21,64]. GtfB from *Streptococcus thermophilus* predominantly introduced branches of DP 1–7 into amylose and DP 1–12 into potato amylopectin, with DP 3 branches being most abundant; this modification reduced iodine affinity, molecular weight, and viscoelasticity while preserving pseudoplastic behavior; it also slowed glucose release by mucosal α-glucosidase and increased SDS content from 34.29% to 53.22% at an enzyme concentration of 5 U/g [65]. Similarly, GtfB from *Lactobacillus reuteri* E81 elevated the α-1,6 linkage ratio in potato starch from 2.9% to 36.8% without markedly compromising granular thermal stability [66].

### 4.4. Amylosucrase

Amylosucrase (EC 2.4.1.4; GH13_4) catalyzes the polymerization of glucose into α-1,4-glucans by harnessing the free energy of the sucrose glycosidic bond, without the requirement for nucleotide sugars or an exogenous primer. Investigations into product distribution and single-molecule dynamics of *Neisseria polysaccharea* amylosucrase have elucidated a “zipper” reaction mechanism, wherein enzyme molecules cooperate along a growing glucan chain, facilitating systematic chain elongation and modulation of digestibility [67].

The most compelling direct evidence involving potato starch pertains to *Deinococcus geothermalis* amylosucrase. Elongation of both waxy and normal potato starches resulted in increased branch-chain lengths, enhanced stabilization of crystalline order, and elevated gelatinization temperatures and peak viscosities. The reaction temperature was found to regulate the balance between elongation and side reactions, yielding slowly digestible starch contents that increased from negligible levels to as high as 19.8%, and resistant starch contents ranging from 38.2% to 62.2%, alongside improved resistance to mammalian mucosal α-glucosidase [68]. Furthermore, recrystallization of amylosucrase-modified waxy potato starch in the presence of added amylose demonstrated that amylopectin chain length governs the formation and digestibility of low-digestible fractions, underscoring the necessity of coordinated design between elongation and retrogradation processes [69].

Other findings should be attributed to their original substrates rather than conflated with potato starch data. For instance, amylosucrase treatment followed by pullulanase digestion converted amylopectin from an unspecified or non-potato source into a low-digestibility product [70]. Additionally, dual modification employing branching enzyme and amylosucrase was demonstrated on sweet potato starch [71], and gel strengthening at low solids content was observed in normal rice starch [72]. Collectively, these studies corroborate the general applicability of elongation and cascade modification concepts but do not replace the need for direct validation using potato starch. The transferases relevant to the modification of potato starch are compiled in Table 2.

### 4.5. Cyclodextrin Glucanotransferase

Cyclodextrin glucanotransferases (CGTases; EC 2.4.1.19; GH13_2) are enzymes belonging to the α-amylase family that specialize in intramolecular transglycosylation. These enzymes catalyze the conversion of starch into α-, β-, and γ-cyclodextrins, which consist of six, seven, and eight glucosyl units, respectively, in addition to facilitating coupling, disproportionation, and limited hydrolysis reactions (Figure 2g) [76]. The architecture of the acceptor site plays a critical role in determining product specificity [36]. Cyclodextrins are capable of forming inclusion complexes with hydrophobic guest molecules and have applications in the food, pharmaceutical, cosmetic, and agrochemical industries [77].

Potato starch serves as an effective substrate for CGTases in both gelatinized and raw granular forms. For instance, the CGTase from *Bacillus macerans* has been shown to degrade intact potato granules, with the interior of the granules appearing more susceptible to enzymatic action than the outer shell [31]. Additionally, a CGTase derived from *Microbacterium terrae* KNR 9 produced 13.46 g/L of cyclodextrins from raw potato starch within one hour at 60 °C. Optimization of reaction conditions using response surface methodology—specifically, 150 g/L starch concentration, 4.8 U/L enzyme activity, and a temperature of 55.6 °C—increased β-cyclodextrin production to 28.22 g/L [73]. It is important to note that these productivity values are specific to the conditions of the respective studies and should not be interpreted as representative of universal industrial yields, as factors such as starch source, granule state, enzyme selectivity, product precipitation, and downstream recovery significantly influence reported titers.

### 4.6. α-Glucan Phosphorylase

α-Glucan phosphorylases (EC 2.4.1.1; GH94) catalyze the reversible phosphorolysis of α-1,4-glucans to produce glucose-1-phosphate. In the synthetic direction, these enzymes facilitate the elongation of primer chains with precise control over molecular weight. The potato L-type α-glucan phosphorylase is among the most extensively characterized members of this family and has been employed in the precision synthesis of amylose as well as in the development of amylose-based hybrid materials [75]. Historically, its practical application was constrained by thermolability; however, the introduction of multiple thermostabilizing mutations has significantly enhanced its thermal stability without compromising catalytic efficiency [74]. Consequently, α-glucan phosphorylase has been reclassified from the established potato-starch modification category to the emerging opportunities subsection, reflecting its primary applications in precision synthesis and materials chemistry rather than in commercial potato-starch food modification [75]. Overall, the most robust direct evidence in potato starch pertains to 4αGT-mediated thermoreversible gelation, GtfB and GBE branching, amylosucrase elongation, and CGTase cyclization. While evidence derived from other starch sources is valuable for elucidating cascade mechanisms and guiding enzyme engineering, it should not be interpreted as directly indicative of potato starch performance.

## 5. Value-Added Products from Enzymatically Modified Potato Starch

### 5.1. Thermoreversible Gelling Starch and Gelatin Alternatives

The benchmark product in potato starch enzymology is amylomaltase-treated gelling starch. The combination of amylose depletion and amylopectin exterior-chain elongation produces a polymer population that, upon cooling, associates into an opaque, elastic, and thermoreversible gel [52,53]. The commercially available product, marketed as Etenia^TM^, has been characterized as a gelling, thickening, and texturizing agent suitable for dairy, bakery, dressing, and vegetarian applications [11]. Its documented capacity to bind whey in fresh cheese and strained yogurt is corroborated by product development literature. The substitution of protein or fat varies depending on the application; the previously asserted universal equivalence—“one part starch replaces one part protein or three parts fat”—has been retracted, as it should not be regarded as a fixed material constant [11,12]. In low-fat yogurt, domains of amylomaltase-treated starch enhanced perceived creaminess relative to maltodextrin controls by mimicking certain aspects of fat-droplet lubrication [12].

Product performance continues to advance: gels derived from *T. uzoniensis* 4αGT exhibit improved freeze–thaw stability [57]; post-treatment with amylosucrase increases gel strength [58]; and controlled residual amylose content allows for tuning of the sol–gel transition temperature [59]. Nonetheless, certain limitations persist: amylomaltase gels are generally opaque rather than transparent, do not fully melt at body temperature—complete melting may require approximately 60 °C—set more slowly than gelatin, and are susceptible to hydrolysis by salivary amylase during consumption [5,19]. Enzyme engineering and the use of enzyme cocktails that more precisely modulate chain-length distributions represent promising strategies to address these challenges. The term “clean-label” functions primarily as a marketing and consumer communication concept rather than a single, harmonized legal standard. Regulation (EC) No 1333/2008 governs food additives and E-numbers within the European Union, whereas Regulation (EU) No 1169/2011 regulates consumer information. In the United States, 21 CFR 172.892 pertains to “food starch-modified” under specified conditions, and enzyme preparations may also be regulated based on their source, function, and Generally Recognized As Safe (GRAS) status. In China, GB 2760-2024 governs permitted food additives. Consequently, an enzyme-modified potato starch may be classified as a clean-label ingredient in one application but as a regulated additive in another; therefore, jurisdiction-, function-, and process-specific evaluations are necessary [7,8,9,10,78].

### 5.2. Resistant and Slowly Digestible Starch Ingredients

Rapidly digestible starch (RDS), slowly digestible starch (SDS), and resistant starch (RS) represent in vitro operational fractions associated with the rate and site of glucose release [16]. Their relationship to physiological outcomes, however, is indirect. Human studies indicate considerable variability in the digestion of potato starch in vivo and demonstrate that consumption of resistant starch can affect glycemic response, satiety, and gut fermentation. Nonetheless, the specific enzyme-modified potato starches reviewed herein have not been consistently evaluated in human trials [79,80,81,82,83]. Consequently, the general health benefits attributed to resistant starch—such as improved glycemic control, enhanced satiety, and gut health—cannot be assumed to apply uniformly to all enzymatically modified potato starch products [4,17,84].

Debranching-based RS3 production methods yield approximately 44–45% resistant starch from potato starch when pullulanase activity and subsequent retrogradation are efficient [44,45]. Transferase-mediated approaches predominate in SDS design, as branching and elongation can delay digestive amylase access while maintaining technological functionality. Three direct potato starch modification strategies exemplify this principle: short α-1,6 branching catalyzed by StGtfB, which increases SDS from 34.29% to 53.22%; broad cluster restructuring via McGBE, resulting in a 65.8% increase in SDS; and exterior-chain elongation by amylosucrase, achieving SDS levels up to 19.8% and RS ranging from 38.2% to 62.2% [62,65,68]. These methods differ in their associated physicochemical properties: StGtfB-modified products exhibit reduced viscoelasticity, McGBE-treated starches demonstrate resistance to retrogradation, and amylosucrase-modified starches can maintain or enhance gelation and thermal stability.

It is important to note that digestibility values are dependent on the analytical methods employed. Techniques such as Englyst kinetics, AOAC total dietary fiber-type RS measurements, hydrolysis indices, predicted glycemic indices, and porcine or human mucosal α-glucosidase assays assess distinct endpoints. Therefore, comparisons of product rankings should be conducted exclusively within the same analytical framework and sample preparation protocols [16,80].

### 5.3. Cyclodextrins and Large-Ring Cycloamyloses

Cyclodextrins are cyclic α-1,4-glucans characterized by hydrophobic cavities that facilitate the solubilization, stabilization, and delivery of lipophilic guest molecules [77]. The production of β-cyclodextrin directly from raw potato starch, achieving a concentration of 28.22 g/L under optimized laboratory conditions, demonstrates that potato starch can circumvent gelatinization within an appropriate CGTase-mediated process [73]. Similarly, γ-cyclodextrin can be synthesized from starch substrates using specific CGTases [85]. Large-ring cycloamyloses, generated through 4αGT-catalyzed cyclization, expand the range of complexable guest molecules and have been proposed as solubilizing agents and facilitators of protein refolding [20,51].

A recent study focusing on direct potato starch processing revealed that the combination of pullulanase hydrolysis and nanoprecipitation enables the production of very small potato starch nanoparticles. Utilizing downgraded potatoes as the raw material, the most favorable conditions reported involved the application of 160 npun pullulanase per gram of starch at 60 °C and pH 4 for 6 h; subsequent nanoprecipitation of the hydrolysates yielded particles approximately 20–50 nm in size. These findings suggest a potential valorization pathway for downgraded potatoes; however, challenges related to nanoprecipitation, ethanol usage, recovery, drying, and compliance with food-grade regulatory standards must be addressed prior to applications in food or pharmaceutical contexts [86]. Representative enzyme-modified potato starch products and their study-specific properties are compiled in Table 3.

Starch nanoparticles and nanocrystals constitute an emerging class of products. Their preparation typically involves a combination of controlled acid or enzymatic hydrolysis with mechanical treatment or recrystallization steps. The specificity of enzymes may contribute to narrowing particle size distributions or preserving particular crystal faces. Nevertheless, much of the existing literature on nanoparticles is not specific to potato starch, and issues concerning scalable recovery methods, the use of food-grade solvents, and regulatory approval remain unresolved. Consequently, starch nanoparticles are classified as emerging rather than established products within the domain of potato starch enzymology [87].

### 5.4. Porous Starch Carriers

Porous starch is produced through the partial hydrolysis of native granules at temperatures below gelatinization, resulting in the formation of surface pores and internal voids while maintaining the structural integrity of the granules [40]. These porous particles are capable of adsorbing oils, flavors, probiotics, and bioactive compounds, thereby functioning as controlled-release matrices. Given that potato starch granules exhibit resistance to enzymatic pitting, the combination of α-amylase or glucoamylase digestion with mild acid hydrolysis has been shown to significantly enhance pore formation in direct experiments involving potato starch [39].

A detailed comparison of pore geometry resulting from α-amylase and glucoamylase treatments was conducted using corn starch; however, the anthocyanin microgel studies did not utilize intact potato granules as the exclusive substrate. Consequently, these investigations serve primarily as mechanistic references. The application of these findings to potato starch for the development of payload-specific carriers warrants further exploration, but it is essential to experimentally verify adsorption capacity, granule integrity, and release behavior rather than extrapolating from results obtained with corn starch [40,88].

### 5.5. Maltodextrins and Glucose Syrups

The classical hydrolase pathway continues to hold commercial significance: thermostable α-amylase-mediated liquefaction of potato starch followed by glucoamylase-driven saccharification produces maltodextrins and glucose syrups with defined dextrose equivalent (DE) values. Limited α-amylase activity results in low-DE maltodextrins utilized as fat mimetics, bulking agents, and in spray-drying applications, whereas prolonged glucoamylase saccharification—often facilitated by pullulanase to hydrolyze α-1,6 glycosidic linkages at limit-dextrin branch points—promotes conversion toward high-DE glucose syrups [41,43]. In comparison to cereal starch processes, potato starch is characterized by high paste viscosity, elevated phosphate content, and large granule size, factors that can affect mixing, liquefaction, filtration, and downstream purification. These well-established bulk applications are primarily maintained to bridge traditional starch processing with emerging enzyme-centric valorization strategies, rather than to position maltodextrins as a novel value-added product [5,18].

Within the product categories, the most robust direct evidence supports thermoreversible gels, debranching-based resistant starch type 3 (RS3), GtfB/GBE slowly digestible starch (SDS) ingredients, amylosucrase-modified low-digestibility starch, cyclodextrins, and acid-assisted porous potato starch. Conversely, nanoparticles, large-ring cycloamyloses beyond laboratory-scale validation, and numerous carrier applications remain at a less advanced stage of development.

## 6. Physical-Field—Enzyme Synergies

**Figure 4:**
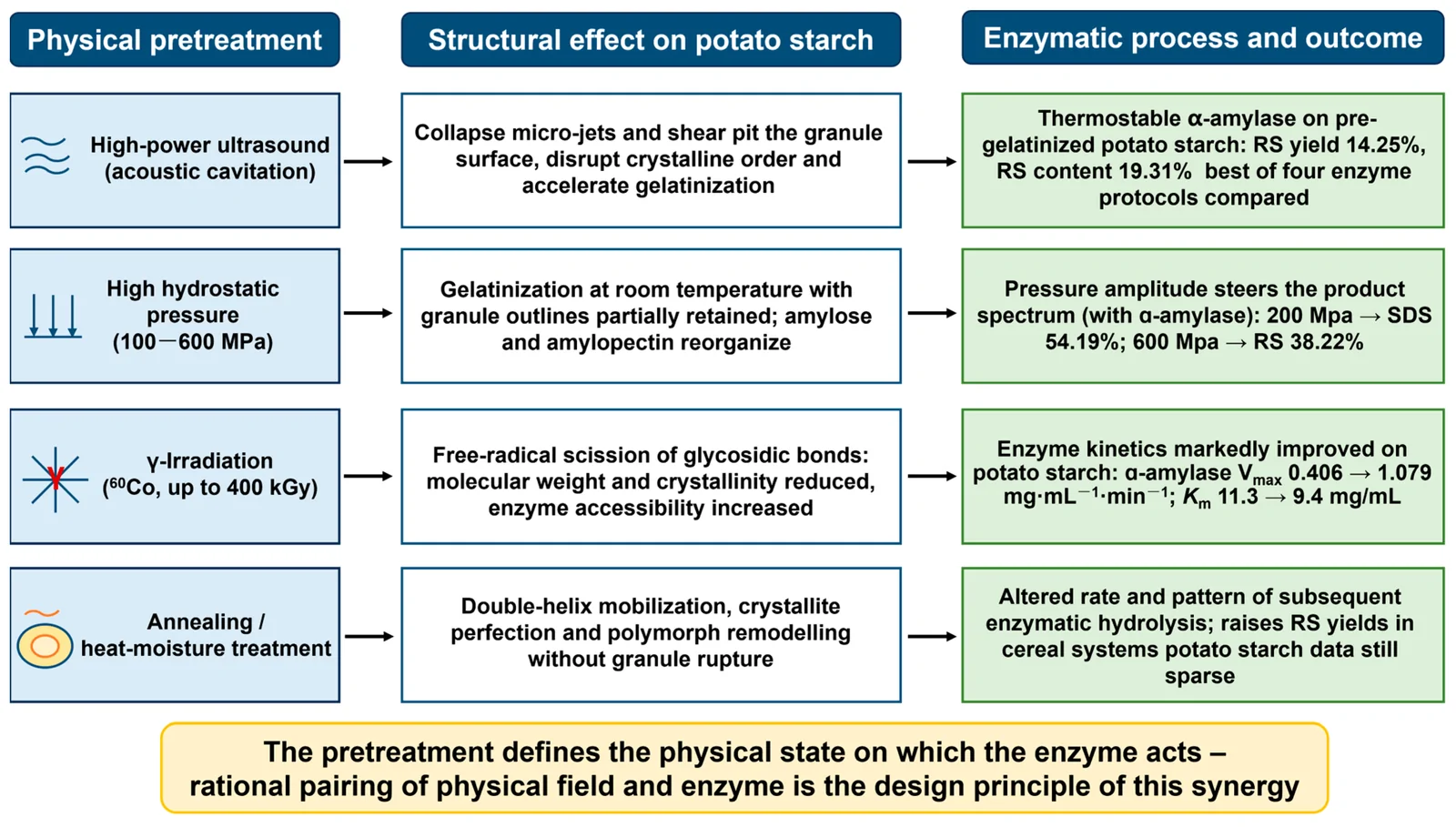
Physical-field pretreatments and their synergistic effects with enzymatic modification are examined. Direct examples involving potato starch are labeled as “potato,” whereas examples pertaining to riceberry, cereal, and generic starches are explicitly identified as extrapolated. High-power ultrasound [49,89], high hydrostatic pressure (HHP) [90,91,92,93], gamma (γ) irradiation [94], and hydrothermal treatments [95,96] each modify granular or molecular accessibility; however, their effects are highly dependent on the specific equipment used and the treatment parameters applied.

Physical pretreatments have the potential to disrupt, swell, or partially depolymerize potato starch, thereby modifying its enzymatic accessibility. Due to the considerable variability in equipment geometry and energy input, parameters such as ultrasound frequency and nominal power, high hydrostatic pressure (HHP) pressure-holding time, irradiation dose, and hydrothermal moisture cannot be standardized by a single universal optimum. Consequently, the examples presented below specify the botanical source and differentiate between direct evidence from potato starch and cross-starch evidence (Figure 4).

### 6.1. Ultrasound

High-power ultrasound induces acoustic cavitation, the collapse of which generates microjets and shear forces capable of pitting granule surfaces, disrupting crystalline structures, and enhancing enzyme accessibility without necessarily introducing chemical substituents [89]. In laboratory studies of starch, low-frequency, high-power probe systems operating approximately within the 20–25 kHz range are commonly employed; however, power, power density, and treatment duration are highly dependent on reactor geometry. These parameters should not be construed as optimal specifically for potato starch [89]. Ultrasound-assisted processing has been shown to improve both the yield and quality of resistant starch derived from pre-gelatinized potato starch using a thermostable α-amylase, achieving the highest resistant starch yield (14.25%) and content (19.31%) among four enzyme-based protocols compared by Cheng et al. [49]. The indexed record included in this review did not allow for independent verification of a universally applicable ultrasound frequency or power setting; therefore, no unverified numerical sonication parameters are proposed herein. The observed synergistic effect is plausible, as cavitation facilitates hydration and gelatinization processes and exposes additional amorphous regions within the starch matrix.

### 6.2. High Hydrostatic Pressure

High hydrostatic pressure (HHP), typically ranging from 100 to 600 MPa, can induce starch gelatinization at moderate temperatures while partially preserving the granule structure, resulting in states that differ from those produced by thermal pasting [90,91]. In the case of potato starch, HHP treatment at 400–600 MPa followed by retrogradation significantly reduced in vitro starch hydrolysis, with the most pronounced effects observed at 600 MPa [92]. Conversely, a study on riceberry starch—rather than potato starch—demonstrated that a 200 MPa pretreatment followed by α-amylase hydrolysis increased slowly digestible starch (SDS) to 54.19%, whereas treatment at 600 MPa shifted the product composition toward 38.22% resistant starch (RS) [93]. These findings illustrate the pressure-dependent modulation of enzymatic hydrolysis products; however, they should not be directly extrapolated to potato starch. Furthermore, parameters such as pressure magnitude, holding time, temperature, decompression rate, and initial moisture content must be clearly defined before applying these results to potato starch systems.

### 6.3. Irradiation

Gamma (γ) irradiation induces depolymerization of starch by generating free radicals that cleave glycosidic bonds, resulting in decreased molecular weight and crystallinity, as well as enhanced enzymatic reactivity. Specifically, potato starch exposed to ^60^Co-γ rays at a dose of 400 kGy exhibited improved kinetic parameters for α-amylase and glucoamylase: the Km value for α-amylase decreased from 11.343 to 9.386 mg/mL, and its Vmax increased from 0.406 to 1.079 mg·mL^−1^·min^−1^; similarly, the Vmax for glucoamylase increased from 0.338 to 0.821 mg·mL^−1^·min^−1^ [94].

Although a 400 kGy dose serves as a useful mechanistic proof of concept, it significantly exceeds the doses typically employed in direct food-starch processing and may induce undesirable effects such as depolymerization, increased acidity, discoloration, or off-flavors. Therefore, this dose should not be considered representative of realistic food-processing conditions. Further research involving lower irradiation doses, control of radiolytic by-products, and adherence to regulatory limits is necessary prior to any food-related applications [94,97].

### 6.4. Annealing and Heat-Moisture Treatment

Annealing, defined as the process of holding starch in excess water at temperatures below the gelatinization onset, facilitates the mobilization of double helices and can enhance the perfection of crystallites without causing granule rupture. In contrast, heat–moisture treatment conducted under restricted moisture conditions can induce remodeling of starch polymorphism [95]. Both treatments influence subsequent enzymatic hydrolysis by altering the relative proportions of amorphous and crystalline regions within the starch. However, direct and systematic data on the effects of annealing–enzyme or heat–moisture–enzyme treatment cascades specifically in potato starch remain limited. Consequently, this approach should be regarded as an underexplored design strategy rather than an established method for potato starch modification. The unique B-type crystalline lattice and high hydration level of potato starch may result in responses that differ from those observed in cereal starches [3,95].

Furthermore, ethanol-solution annealing has been demonstrated to modify the physicochemical properties of potato starch, including chain reorganization and hydration behavior [96]. Although this investigation did not involve an enzymatic cascade, it supports the potential for solvent-controlled annealing to modulate the granular state prior to enzymatic treatment. Studies directly comparing the effects of annealing intensity, enzyme identity, and treatment sequence on potato starch remain scarce and should be prioritized as a significant research gap rather than considered an established process.

The application of physical fields can reduce enzyme requirements or direct product profiles; however, these methods introduce additional capital and operational complexities. Techniques such as ultrasound and high hydrostatic pressure (HHP) necessitate energy-intensive, specialized equipment; irradiation poses regulatory challenges and may affect consumer acceptance; and hydrothermal treatments demand precise control of moisture and temperature. Therefore, any claims of synergistic effects should be substantiated by comprehensive evaluations encompassing product yield, enzyme dosage, solids concentration, energy consumption, water usage, and downstream recovery, rather than relying solely on measures of enzyme activity. Representative process and kinetic outcomes discussed in this review are compiled in Table 4.

## 7. Enzyme Engineering for Tailored Potato Starch Modification

The enzymes discussed above were originally identified primarily through the screening of natural diversity. Currently, protein engineering serves as a complementary approach to discovery by enhancing enzyme stability, specificity, and compatibility with industrial processes.

Thermostability engineering has been achieved in bacterial α-amylases via strategies such as surface-charge optimization, loop rigidification, and the introduction of disulfide bonds or salt bridges, guided by the conserved Ca^2+^-binding architecture characteristic of this enzyme family [98]. For example, structure-based design of the alkaline α-amylase from *Alkalimonas amylolytica* yielded the H209L/Q226V/P477V variant, which exhibited a 3.8-fold increase in half-life at 60 °C, a 3.2 °C elevation in melting temperature, and a 24% enhancement in specific activity [99]. A single amino acid substitution, E27R, improved both alkaline and thermal stability in *Thermus filiformis* amylomaltase [100], while cumulative mutations enhanced the stability of potato L-type α-glucan phosphorylase [74]. In cases where structural information is limited, consensus design and directed evolution remain valuable methodologies [101,102]. It is noteworthy that maximal thermostability is not invariably advantageous; for instance, moderate stability of Um-αAmy permitted termination of processing at a desired depolymerization endpoint [15].

Specificity and subsite engineering have been advanced through structure-guided mutagenesis, which has delineated acceptor subsites +2 and +3 in *Thermus thermophilus* amylomaltase [103], and identified the influence of the 250s loop on substrate recognition in *Thermus brockianus* amylomaltase [104]. These structural insights provide a foundation for reprogramming donor and acceptor preferences, thereby modulating disproportionation, cyclization, or hydrolysis outcomes. Through CUPP-guided mining of over 12,000 GH77 sequences, 27 representative enzymes were identified with a high success rate for potato starch modification [56]. Additionally, multivariate fingerprinting of twenty GH13 α-amylases clustered these enzymes into six functional groups correlated with active-site openness [15,38]. Collectively, these studies support a preliminary framework linking sequence groups to active-site geometry and ultimately to product fine structure; however, this framework remains predictive rather than universally quantitative.

Process engineering necessitates alignment of enzyme performance with practical industrial conditions. While immobilization can facilitate catalyst recovery, it may concurrently diminish activity against high-molecular-weight potato starch and complicate mass transfer. Enzyme cascades, such as branching enzymes followed by 4αGT or amylosucrase followed by pullulanase, enable access to structures unattainable by single enzymes [63,70,71]. Nonetheless, successful industrial implementation depends on factors beyond catalytic efficiency, including the cost and dosage of food-grade enzymes, production consistency, thermostability, reaction time, starch solids concentration and viscosity, energy requirements for gelatinization and cooling, water consumption, downstream washing or fractionation, product drying, enzyme recovery or inactivation, scale-up challenges related to mixing and heat transfer, and co-product valorization. Many laboratory studies employ dilute starch systems and extended incubation periods; consequently, achieving efficient conversion at industrially relevant solids concentrations, enzyme turnover rates, and overall process cost remains a significant scale-up challenge [5,49].

**Figure 5:**
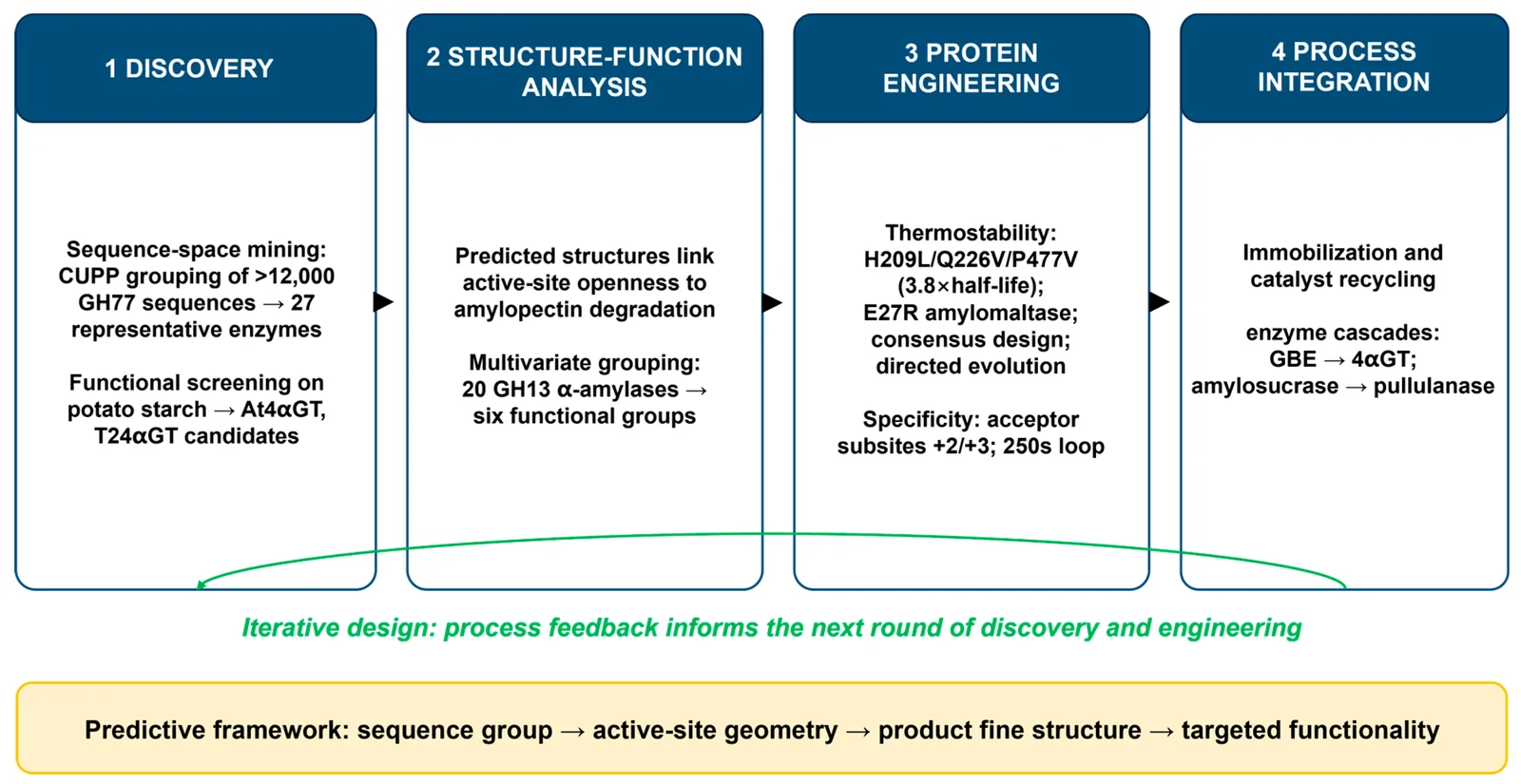
The enzyme discovery and protein engineering pipeline for customized modification of potato starch involves several key stages. Candidate enzymes are identified through sequence-space mining and functional screening [56]. Structural prediction methods are employed to correlate active-site geometry with the fine structure of the resulting products [15,38]. Subsequently, rational engineering and directed evolution techniques are applied to enhance enzyme stability and specificity [98,99,100,101,102,103,104]. Finally, process integration facilitates the development of industrial biocatalysts [35,105]. Recent advancements in this field include the incorporation of cold-active enzymes, interfacial catalysis, and multiple functionalization strategies [106].

Cold-active and psychrotolerant enzymes represent an alternative strategy to reduce process energy consumption. Recent investigations into cold-active enzymes derived from lactic acid bacteria underscore their potential for catalysis at reduced temperatures, which may be advantageous for processing temperature-sensitive foods or for minimizing excessive gelatinization during potato starch processing [105]. However, their applicability to concentrated and highly viscous potato starch systems has yet to be established, as low temperatures can concurrently impede hydration, chain mobility, and mass transfer. The enzyme discovery and protein-engineering pipeline integrating these strategies is summarized in Figure 5.

## 8. Comparative, Regulatory and Industrial Assessment

### 8.1. Comparison of Chemical, Physical, and Enzymatic Modifications

Chemical modification techniques are well-established, rapid, and effective at high solids concentrations, supported by extensive industrial application and regulatory experience. These methods enable the introduction of cross-links, substituents, or oxidized groups with high process reliability. However, potential drawbacks include residual reagents, by-product streams, consumer perception issues, and additive labeling requirements. In contrast, physical modification methods avoid the use of chemical reagents but often necessitate the application of heat, moisture control, shear, pressure, or irradiation, which may result in less specific molecular alterations. Enzymatic modification offers high molecular specificity under relatively mild processing conditions but typically requires longer reaction times, higher enzyme dosages, dilute systems, and subsequent enzyme inactivation or separation. A recent comprehensive review of starch functionalization highlights that the sequence and intensity of physical, chemical, and enzymatic treatments are as critical as the number of treatments applied; ostensibly similar treatment combinations can yield divergent outcomes due to factors such as botanical origin, initial crystallinity, chain architecture, and cumulative process severity [106]. Consequently, potato starch should not be considered an interchangeable substitute for maize, rice, or tapioca starch in dual- or multiple-modification protocols.

### 8.2. Industrial Application and Relevance to China

China is the world’s largest producer of fresh potatoes, with recent FAOSTAT data indicating annual production in the range of 90–100 million tonnes, accounting for approximately one quarter of global output [1]. While fresh potato tonnage is consistently documented, data on industrial potato starch volume and value are fragmented across provinces and processing entities. Potato processing is concentrated in major producing and processing regions, including northern, northwestern, and southwestern China, and faces challenges such as seasonal raw material supply, dispersed processing capacity, high water and drying energy demands, starch-loss control, and valorization of process streams. Enzymatic modification has the potential to enhance starch functionality and upgrade side streams; however, its industrial advantages must be demonstrated relative to established chemical and physical processes, considering local raw materials, energy costs, and wastewater management constraints.

Priorities specific to China’s industrial context include direct validation on locally cultivated potato varieties and industrial potato starch; cost-effective production or procurement of food-grade enzymes; development of high-solids reactors capable of processing highly viscous pastes; integration with efficient gelatinization, liquefaction, and drying processes; reuse of process water; and compliance with GB 2760 additive and labeling regulations. The enzymatic methodologies reviewed herein can support these objectives, but commercial claims should be substantiated through Chinese pilot-scale trials rather than solely laboratory-scale data.

### 8.3. Regulatory and Life-Cycle Considerations

Regulatory requirements vary by jurisdiction. In the European Union, additive authorization and labeling are governed by Regulation (EC) No 1333/2008 and Regulation (EU) No 1169/2011. In the United States, modified food starch is regulated under 21 CFR 172.892, with enzyme preparations subject to Generally Recognized as Safe (GRAS) status and processing-aid regulations. In China, GB 2760-2024 and associated standards regulate permitted additive use. Canada maintains positive lists of authorized food additives [7,8,9,10,78].

Comparative enzyme-specific life-cycle assessments (LCAs) for potato starch modification remain limited. A robust LCA should adhere to ISO 14040/14044 standards and encompass all relevant stages, including potato cultivation or starch procurement, gelatinization, enzyme production, reaction, separation or inactivation, water recycling, drying, and co-product allocation [107,108]. Currently, assertions regarding the environmental superiority of enzymatic modification routes should be regarded as plausible hypotheses rather than established conclusions.

## 9. Limitations of This Review

This review employs a structured narrative approach rather than a PRISMA-compliant systematic review, primarily due to the pronounced heterogeneity in outcome measures, experimental designs, and reporting conventions across the evidence base. Consequently, certain potentially relevant sources—including patents, theses, and regionally published studies—may have been inadvertently omitted because of limitations in database coverage, indexing practices, and citation tracking. Reporting of process parameters for ultrasonication and other physical-field pretreatments is often incomplete or inconsistent across the available literature; such instances are explicitly noted in the “Evidence and Caution” column of Table 4. Furthermore, the quantitative yield and digestibility data compiled here were obtained from studies employing diverse starch cultivars, substrate states, reaction conditions, and assay methodologies, which inevitably limits direct cross-study comparisons. Finally, a substantial portion of the mechanistic insights regarding active-site architecture, carbohydrate-binding modules, pore geometry, and nanoparticle formation are derived from non-potato starches, so their extrapolation to potato starch should be interpreted with appropriate caution. Taken together, these limitations underscore the necessity of explicitly distinguishing direct evidence from extrapolated evidence, as implemented throughout this review, and highlight the imperative of establishing standardized reporting protocols specific to potato starch.

## 10. Conclusions and Future Perspectives

Potato starch occupies a unique niche within the domain of enzymatic starch modification. Its large, phosphorylated, B-type crystalline granules exhibit resistance to enzymatic attack in their native granule form, whereas gelatinized potato starch demonstrates pronounced responsiveness to modifications in chain length and glycosidic linkages. Empirical evidence substantiates the formation of thermoreversible gels, pullulanase-assisted resistant starch type 3 (RS3), GtfB/GBE-mediated slowly digestible starch (SDS), amylosucrase-modified low-digestibility starch, cyclodextrin production, and acid-assisted porous starch. Quantitative benchmarks assembled in this review illustrate the scale of these effects: enzymatic modification can raise the resistant starch (RS) yield of potato starch to 44.48 ± 1.37% (combined α-amylase–pullulanase treatment), increase the SDS fraction from 34.29% to 53.22% (StGtfB), and elevate the α-1,6 linkage content from 3.0% to 6.0% (McGBE), while γ-irradiation pretreatment (400 kGy) increases the V_max_ of α-amylase toward potato starch by a factor of approximately 2.7, underscoring how strongly physical pretreatment can amplify enzymatic action. Although advancements have been made toward structure-guided enzyme selection for 4-α-glucanotransferases (4αGTs) and α-amylases, it is important to recognize that these developments do not yet constitute a comprehensive predictive design framework applicable to all starch-active enzymes. Bridging this gap will require the integration of high-throughput enzyme mining (e.g., CUPP-based screening), machine-learning-assisted structure–function prediction, and standardized reporting of reaction outcomes, as outlined in the priority list below.

High Priority (approximately 0–3 years):

1. Develop and validate raw-granule-active transferases or carbohydrate-binding module (CBM)-equipped enzymes targeting intact potato starch to reduce or obviate the energy-intensive gelatinization step.

2. Establish standardized potato starch substrates, reaction conditions, resistant starch (RS) and slowly digestible starch (SDS) assays, and reporting protocols encompassing both yield and product composition.

3. Directly evaluate enzyme cascades—such as branching enzyme followed by 4αGT, and amylosucrase followed by debranching enzyme—on potato starch.

Medium Priority (approximately 3–5 years):

4. Extend structure-guided engineering efforts beyond 4αGTs and α-amylases to include GtfB, glucan branching enzyme (GBE), and cyclodextrin glycosyltransferase (CGTase), accompanied by experimental validation of predicted donor and acceptor specificities.

5. Integrate physical treatments such as ultrasound, high hydrostatic pressure (HHP), hydrothermal processing, and irradiation with enzyme selection at industrially relevant solid concentrations, incorporating assessments of energy and water balances.

6. Develop pilot-scale processes for product recovery, enzyme inactivation, and downstream fractionation. Longer-Term Priority (approximately 5–10 years):

7. Conduct product-specific human clinical studies assessing glycemic response, satiety, and gut fermentation, moving beyond reliance on generic resistant starch evidence.

8. Complete ISO-compliant life-cycle and techno-economic assessments within major processing regions, including China.

9. Clarify jurisdiction-specific regulatory statuses and consumer labeling requirements for each commercially available enzyme-modified potato starch product.

The principal design paradigm emerging from this review emphasizes that enzyme selection must be aligned sequentially with the physical state of potato starch, the desired fine structural attributes, and finally, process and regulatory considerations. This substrate-centered framework constitutes the principal contribution of the present enzyme-focused synthesis. Looking forward, the convergence of enzyme discovery, protein engineering, physical-field pretreatment, and life-cycle-informed process design is expected to transform potato starch from a commodity hydrocolloid into a family of precision-functionalized ingredients with tunable digestibility, texture, and delivery properties. Realizing this vision will depend on close collaboration among enzymologists, food scientists, process engineers, and regulatory bodies, as well as on the establishment of open structure–function databases specific to potato starch. We anticipate that the framework and evidence base assembled here will accelerate the rational, sustainable production of next-generation potato starch ingredients for both food and non-food applications.

## Figures and Tables

**Table 1:** Hydrolases applied to the modification or analysis of potato starch.

Enzyme (EC; GH Family)	Representative Source	Substrate State and Condition	Catalytic Action	Main Product or Effect	Potato-Starch Evidence	References
α-Amylase (EC 3.2.1.1; GH13)	*Bacillus licheniformis*; Um-αAmy	Gelatinized or pasted starch for gel tailoring; granular starch for surface erosion	Endo-hydrolysis of α-1,4 bonds; action pattern controls depolymerization	Liquefaction, maltodextrins, porous starch, tailored gelling starches	Direct for Um-αAmy and potato porous starch; extrapolated for generic liquefaction enzymes	[15,18,37,39]
β-Amylase (EC 3.2.1.2; GH14)	*Bacillus cereus*; soybean	Raw potato granules; solubilized chains for chain profiling	Exo-release of maltose; stops at branch points	Maltose; β-limit dextrin; analytical chain information	Direct for slow raw-granule attack; mainly analytical	[13]
Glucoamylase (EC 3.2.1.3; GH15)	*Aspergillus niger*; *Rhizopus oryzae*	Granular potato starch under subgelatinization conditions; hydrolysates for saccharification	Exo-release of β-glucose; CBM20 supports raw-starch binding	Porous starch in combination with α-amylase; glucose syrups	Direct for potato porous-starch process; CBM mechanism partly extrapolated	[39,41,42]
Pullulanase/isoamylase (EC 3.2.1.41/3.2.1.68; GH13)	Hot-spring metagenome PulM; industrial pullulanases	Gelatinized potato starch, followed by retrogradation; intact granules for isoamylase studies	α-1,6 branch-point cleavage; supplies linear chains	RS3; reported potato RS yield up to approximately 44–45% under study-specific conditions	Direct	[43,44,45,46]

**Table 2:** Transferases relevant to potato starch modification.

Enzyme (EC; GH Family)	Representative Source	Substrate State and Condition	Catalytic Action	Main Product or Effect	Potato-Starch Evidence	References
4-α-Glucanotransferase (EC 2.4.1.25; GH77/GH13_5)	*Thermus thermophilus*; *Pyrobaculum aerophilum*; *Thermoproteus uzoniensis*	Gelatinized/pasted potato starch; typically 60–95 °C depending on enzyme	Disproportionation; amylose depletion and exterior-chain elongation; cyclization	Thermoreversible gels; freeze–thaw-stable gels; cycloamylose	Direct and comparatively strong	[14,52,56,57,59]
Glycogen branching enzyme (EC 2.4.1.18; GH13_8/9)	*Geobacillus thermoglucosidans*; *Microvirga* sp. MC18	Gelatinized or granular starch, depending on process	α-1,4 cleavage and α-1,6 transfer; shortened, denser clusters	Anti-retrogradation starch; SDS enrichment	Direct for McGBE on potato starch	[61,62]
4,6-α-Glucanotransferase GtfB (GH70)	*Streptococcus thermophilus*; *Lactobacillus reuteri* E81	Gelatinized/pasted potato starch; study-specific enzyme dosage	α-1,4 cleavage and α-1,6 transfer; short branches	SDS enrichment; low-viscoelastic starch; increased α-1,6 ratio	Direct	[65,66]
Amylosucrase (EC 2.4.1.4; GH13_4)	*Neisseria polysaccharea*; *Deinococcus geothermalis*	Gelatinized potato starch plus sucrose; temperature-dependent elongation	Sucrose-powered α-1,4 chain elongation	Low-digestibility starch; enhanced gelation and thermal stability	Direct for potato starch; rice/sweet-potato evidence separated	[67,68]
CGTase (EC 2.4.1.19; GH13_2)	*Bacillus macerans*; *Microbacterium terrae* KNR 9	Raw or gelatinized potato starch; up to about 60 °C in reported process	Intramolecular transglycosylation; cyclization	α-, β- and γ-cyclodextrins	Direct, including raw granules	[31,73]
α-Glucan phosphorylase (EC 2.4.1.1; GH94)	Potato L-type enzyme	Primer-dependent synthetic reactions; mainly laboratory precision synthesis	Reversible phosphorolysis and controlled chain elongation	Precision amylose and hybrid materials	Emerging rather than established product evidence	[74,75]

**Table 3:** Representative enzyme-modified potato starch products and study-specific properties.

Product	Enzyme/Process	Key Functional Outcome	Evidence Class	References
Thermoreversible gelatin-like gel	*P. aerophilum* amylomaltase	Gel at ≥3% starch; transition over approximately 37–60 °C	Direct	[5,52]
Commercial gelling/texturizing ingredient	*T. thermophilus* amylomaltase	Gelling/texturizing; whey binding in selected dairy applications	Direct product evidence	[11]
Low-fat yoghurt texturizer	Amylomaltase-treated starch domains	Improved creaminess relative to maltodextrin control	Direct model food evidence	[12]
Freeze–thaw-stable gel	*T. uzoniensis* 4αGT, optionally followed by amylosucrase	Improved freeze–thaw stability and gel strength	Direct	[57,58]
High-SDS potato starch	*S. thermophilus* GtfB, 5 U/g	SDS 34.29% → 53.22%; lower viscoelasticity	Direct	[65]
α-1,6-enriched potato starch	*L. reuteri* E81 GtfB	α-1,6 linkage ratio 2.9% → 36.8%	Direct	[66]
Anti-retrogradation SDS starch	*Microvirga* sp. MC18 GBE	SDS increased by 65.8%; short-chain fraction increased	Direct	[62]
Potato RS3	PulM debranching and retrogradation	Apparent amylose increased by about 80%; approximately 45% RS3	Direct	[44]
Potato resistant starch	α-Amylase followed by pullulanase	RS yield 44.48 ± 1.37%; RDS 21.23%; SDS 36.32%	Direct	[45]
Ultrasound-assisted potato RS	Ultrasound plus thermostable α-amylase	RS yield 14.25%; RS content 19.31%	Direct	[49]
Low-digestibility potato starch	*D. geothermalis* amylosucrase	SDS up to 19.8%; RS 38.2–62.2%	Direct	[68]
β-Cyclodextrin	*M. terrae* CGTase on raw potato starch	28.22 g/L under optimized laboratory conditions	Direct	[73]
Porous potato starch	α-Amylase or glucoamylase plus mild acid	Enhanced pore formation in resistant B-type granules	Direct for potato; pore geometry partly extrapolated	[39,40]
Potato starch nanoparticles	Pullulanase hydrolysis and nanoprecipitation	Approximately 20–50 nm particles under reported conditions	Direct emerging evidence	[86]
Large-ring cycloamylose/nanoparticles	4αGT cyclization or controlled enzymatic erosion	Emerging complexation and delivery applications	Emerging; often non-potato evidence	[51,87]

Note: The numerical values reported were derived under varying conditions, including different starch concentrations, enzyme dosages, reaction durations, product definitions, and analytical methodologies. Consequently, these values should be interpreted as study-specific findings rather than directly comparable industrial standards.

**Table 4:** Representative process and kinetic outcomes discussed in this review.

System	Starch and Physical State	Process Condition	Study-Specific Outcome	Evidence and Caution	References
Irradiated potato starch + α-amylase	Potato; native and 400 kGy-irradiated	Michaelis–Menten assay	*K*_m_ 11.343 → 9.386 mg/mL; Vmax 0.406 → 1.079 mg·mL^−1^·min^−1^	Direct; 400 kGy is mechanistic, not a practical food dose	[94]
Irradiated potato starch + glucoamylase	Potato; native and 400 kGy-irradiated	Michaelis–Menten assay	Vmax 0.338 → 0.821 mg·mL^−1^·min^−1^	Direct; same dose caution	[94]
PulM debranching	Gelatinized potato starch	Optimum 40 °C; >50% residual activity at 4 °C; retrogradation	Apparent amylose + 80%; approximately 45% RS3	Direct	[44]
α-Amylase–pullulanase RS3	Gelatinized potato starch	α-Amylase: pH 6.5, 70 °C, 15 min, 4 U/g; pullulanase: pH 5.0, 60 °C, 24 h, 8 U/mL	RS yield 44.48 ± 1.37%; RDS 21.23%; SDS 36.32%	Direct	[45]
Isoamylase-treated granular potato starch	Intact potato granules	40% *w*/*w*; pH 4.5; 35 °C; 60 min; 100–400 μL of 200 U/mL enzyme per 50 g starch	Predominant amylopectin debranching with retained granular integrity	Direct; process-blank controls needed	[46]
Ultrasound–α-amylase RS	Pre-gelatinized potato starch	Ultrasound-assisted thermostable α-amylase protocol	RS yield 14.25%; RS content 19.31%	Direct; sonication settings not generalized without verified full-process data	[49]
StGtfB	Potato starch	5 U/g	SDS 34.29% → 53.22%	Direct	[65]
McGBE	Potato starch	GBE treatment under study-specific conditions	SDS + 65.8%; α-1,6 content 3.0% → 6.0%	Direct	[62]
*D. geothermalis* amylosucrase	Waxy or normal potato starch	Sucrose-powered elongation; temperature-dependent	SDS up to 19.8%; RS 38.2–62.2%	Direct	[68,69]
*M. terrae* CGTase	Raw potato starch	150 g/L starch, 4.8 U/L, 55.6 °C	β-CD 28.22 g/L	Direct laboratory optimization	[73]
HHP–α-amylase	Riceberry starch, not potato	200 or 600 MPa pretreatment	SDS 54.19% or RS 38.22%	Extrapolated	[93]

## Data Availability

No new data were generated or analyzed in this review. Data sharing is not applicable to this article.

## Abbreviations

4αGT, 4-α-glucanotransferase; αGP, α-glucan phosphorylase; CBM, carbohydrate-binding module; CD, cyclodextrin; CGTase, cyclodextrin glucanotransferase; DE, dextrose equivalent; EFSA, European Food Safety Authority; FDA, U.S. Food and Drug Administration; GBE, glycogen branching enzyme; GH, glycoside hydrolase; HHP, high hydrostatic pressure; LCA, life-cycle assessment; RDS, rapidly digestible starch; RS, resistant starch; SDS, slowly digestible starch.
